# Editorial: Insights into mechanisms underlying brain impairment in aging, volume II

**DOI:** 10.3389/fnagi.2023.1242271

**Published:** 2023-07-11

**Authors:** Jolanta Dorszewska, Kevin T. Ong, Matthew Zabel, Cristina Marchetti

**Affiliations:** ^1^Laboratory of Neurobiology, Department of Neurology, Poznan University of Medical Sciences, Poznań, Poland; ^2^Armadale Health Service, Mount Nasura, WA, Australia; ^3^University of California, Santa Cruz, Santa Cruz, CA, United States; ^4^Institute of Molecular Biology and Pathology (IBPM), National Research Council, Rome, Italy; ^5^European Brain Research Institute (EBRI)-Fondazione Rita Levi-Montalcini, Rome, Italy

**Keywords:** neurodegeneration, aging, molecular factors, neuroinflammation, cerebrovascular diseases

This issue is a continuation of our publication entitled *Insights into mechanisms underlying brain impairment in aging*, Editors: Jolanta Dorszewska, Kevin T. Ong, Matthew Zabel, Cristina Marchetti, published in: Frontiers in Aging Neuroscience in 2021.

Neurodegenerative diseases still pose a serious diagnostic and therapeutic challenge. Moreover, their pathogenesis is not fully understood. The mechanisms leading to neuronal damage include oxidative stress, disorders of the immune response, and the presence of certain genetic variants (Dorszewska et al., 2007; Kowalska et al., 2020; Korczowska-Ła̧cka et al., 2023). In addition, the treatment of neurological patients remains a challenge. There are no effective drugs to relieve and prevent clinical symptoms in patients with common neurodegenerative diseases such as Alzheimer's disease (AD), Parkinson's disease (PD), and Huntington's disease (HD). The current issue in “Frontiers in Aging Neuroscience” deals primarily with the basics of the pathogenesis of neuronal damage and indicates the involvement of both biochemical and genetic factors in the clinical manifestation of neurological diseases. In this issue, we also indicate the need to continue research into the cause of their occurrence.

The number of people over 60 is increasing worldwide. According to modern knowledge, the aging process contributes to a decrease in the efficiency of defense and repair systems and leads to subclinical changes in the central nervous system, including morphological and functional deterioration of brain function, progressive loss of neurons, reduced levels of neurotransmitters, excessive inflammation, impaired integrity vascular, neuroplasticity disorders, the influence of infectious agents, and increase in pathological proteins (Marchetti and Marie, 2011; Dorszewska, 2013; Dorszewska et al., 2016, 2020, 2023; Kozubski et al., 2021; Piekut et al., 2022) (Figure 1).

**Figure 1:**
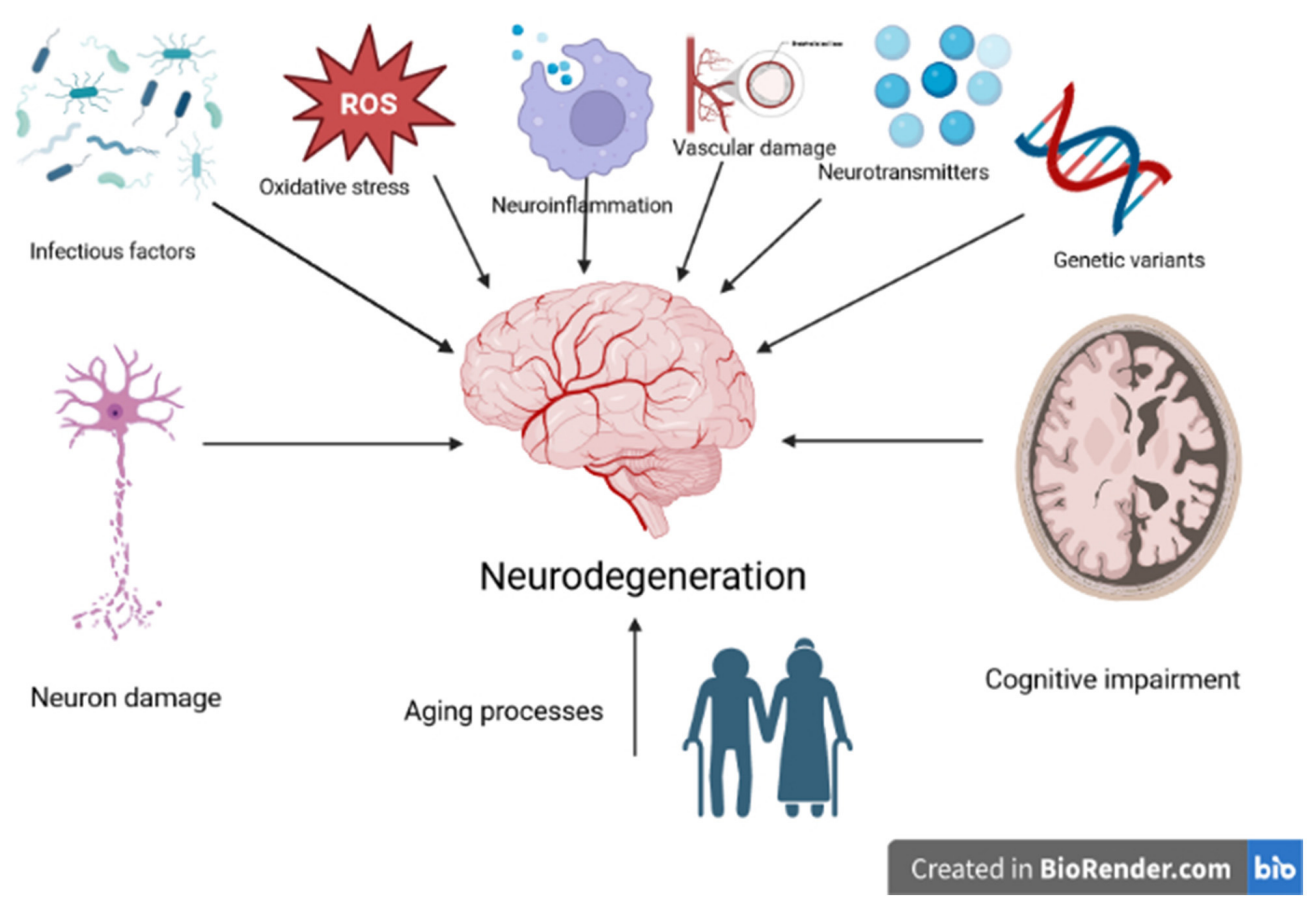
Factors affecting the development of neurodegeneration.

In addition, in the aging brain, the declining effectiveness of repair mechanisms increases susceptibility to reactive oxygen species, neuroinflammatory processes, and spontaneous mutagenesis leading to the development of age-related diseases, including dementia, depression, AD, PD, stroke, epilepsy and other neurodegenerative diseases (Dorszewska et al., 2007, 2021; Dorszewska, 2013; Prendecki et al., 2018, 2019; Kowalska et al., 2020; Korczowska-Ła̧cka et al., 2023) (Figure 1).

In this issue, we address the cause of the primary clinical manifestation, which is cognitive dysfunction in the elderly. As is well known, cognitive decline remains very underdiagnosed. It appears that machine learning-based EEG testing may offer a non-invasive, low-cost approach to identifying cognitive decline. In studies conducted using EEG in 60 elderly people in various cognitive states (MMSE scale) and 22 healthy people from the control group, it was shown that MMSE results significantly correlated with reaction time and EEG A0 and ST4 features. In addition, EEG features showed increased Theta, Delta, A0, and VC9 activity only in young participants, indicating a different pattern of activity between young and older participants in different cognitive states. Future studies should explore the potential usefulness of this tool to characterize cognitive changes on a large scale in a clinical setting to enable early diagnosis of cognitive decline and features of dementia.

It is known that aging-related cognitive decline is related to brain structural changes and loss of synapses (Dorszewska, 2013), but may simultaneously be gender-specific. In this issue, we describe using a model from GTEx transcriptomic data from 13 brain regions of gene co-expression networks and aging-related modules and key regulators common to both sexes and specific to men and women. We indicate that the hippocampus and hypothalamus are more sensitive in males, while the cerebellar hemisphere and anterior cingulate cortex are more at risk of developing neurodegeneration in females. Furthermore, aging-related genes in the hippocampus and frontal cortex appear to be more frequently involved in the pathogenesis of AD. It seems that the development of AD in particular sexes may be conditioned by a diverse molecular background.

Scientific research into whether microorganisms can cause neurodegeneration goes back over 30 years. Many theories have been put forward as to how microorganisms can lead to the development of neurodegeneration, ranging from their ability to interfere with protein trafficking and cause protein aggregation, the activation of microglia toward a pro-inflammatory phenotype consistent with poorer clearance of cellular debris, and a number of other processes (Piekut et al., 2022; Dorszewska et al., 2023).

In this issue, we also report on the potential involvement of microorganisms in the pathogenesis of PD and stroke. In PD, the bacterial microbiota is directly related to parameters that play a potential role in normal intestinal integrity, immunity, metabolism, and proper functioning of the gut-brain axis. We suggest that the gut microbiota may be used for early diagnosis of PD and potential targets for preventing or treating the symptoms of this disease. Furthermore, preserving intestinal homeostasis with probiotics may lead to a promising treatment strategy for PD. One from them, the Symprove™ probiotic, which significantly improves the parameters of both the intestines and the brain in the experimental PD model may be used in the prevention of PD in the future.

At the same time, dysbiosis resulting from a change in the composition and function of the intestinal microflora may cause disturbances in the microbiota-gut-brain axis and be the beginning of stroke and closely associated with the development of stroke risk factors. Summing, further research on the participation of microorganisms in the development of diseases based on neuronal degeneration may contribute to improving the diagnosis and treatment of incurable neurological diseases.

## Conclusion

It seems that a better understanding of the molecular mechanisms associated with neurodegeneration may contribute to a better understanding of the causes of sudden neuronal death in specific areas of the central nervous system. Understanding the mechanism of neuronal damage may contribute to slowing down the progression or delaying the onset of degenerative neurological diseases and to improving the quality of life of people affected by a neurodegenerative disease.

## Author contributions

All authors listed have made a substantial, direct, and intellectual contribution to the work and approved it for publication.
